# Biochemical Characterization of Diverse Stat5b-Binding Enhancers That Mediate Growth Hormone-Activated Insulin-Like Growth Factor-I Gene Transcription

**DOI:** 10.1371/journal.pone.0050278

**Published:** 2012-11-20

**Authors:** Ben Varco-Merth, Kasim Mirza, Damir T. Alzhanov, Dennis J. Chia, Peter Rotwein

**Affiliations:** 1 Department of Biochemistry and Molecular Biology, Oregon Health & Science University, Portland, Oregon, United States of America; 2 Department of Pediatrics, Mt. Sinai School of Medicine, New York, New York, United States of America; Wayne State University, United States of America

## Abstract

Many of the biological effects of growth hormone (GH) are mediated by insulin-like growth factor I (IGF-I), a 70-amino acid secreted peptide whose gene expression is rapidly induced by GH via the Stat5b transcription factor. We previously identified multiple evolutionarily conserved GH-activated chromosomal binding domains for Stat5b within the rat *Igf1* locus, and proposed that they could regulate IGF-I gene activity. Here we investigate the biochemical and functional characteristics of these putative long-range transcriptional enhancers. Each element contained 2 or 3 individual Stat5b recognition sequences that could bind Stat5b *in vitro*, but with affinities that varied over a >100-fold range. Full transcriptional responsiveness to GH required that all Stat5b sites be intact within an individual enhancer. Replacement of a single lower-affinity Stat5b sequence with a higher-affinity one increased *in vitro* binding of Stat5b, and boosted transcriptional potency of the entire element to GH. As enhanced transcriptional activity involved changes in only one or two nucleotides within an enhancer DNA segment, there appears to be remarkable specificity and sensitivity in the ability of Stat5b to transform DNA binding activity into transcriptional function. Stat5b was able to stimulate the transcriptional activity of two enhancers in the absence of GH, indicating that individual Stat5b-regulated elements possess distinct functional features. We conclude that combinatorial interplay among multiple Stat5b-binding response elements with distinguishable biochemical properties is responsible for highly regulated control of IGF-I gene activity by GH.

## Introduction

Growth hormone (GH) plays a pivotal role in multiple physiological processes in mammals. It is essential for somatic growth, is a key contributor to normal tissue differentiation and repair, and is an important regulator of intermediary metabolism [1], [2]. GH also has been implicated in aging and in the development of certain cancers [1], [3]–[8], implying that in the adult its activity must be limited in scope and duration to maintain physiological homeostasis. Thus, it is important to understand mechanisms of GH action in order to devise strategies to enhance its positive physiological effects while limiting its negative impact on human disease.

Like other members of the cytokine receptor family, upon ligand binding the GH receptor engages and stimulates the Jak - Stat signaling pathway [7], [9]–[11]. GH binding induces the receptor-associated tyrosine kinase, Jak2 [7], [9] to phosphorylate tyrosine residues on the intracellular part of the receptor [1], [8], [12], leading to the recruitment of several Stats, as well as other signaling molecules [1], [8], [12].

Stats comprise a group of seven related proteins in mammals [7], [9]–[11], with the first members being characterized as signaling agents for interferons α/β and γ [13], [14]. Subsequent studies have broadened the biological importance of this protein family as critical components of multiple physiological and patho-physiological processes [7], [9]–[11]. Stats are typically found in the cytoplasm of responsive cells prior to hormone or cytokine stimulation. After being recruited to phosphorylated tyrosine residues on intracellular segments of activated receptors, they become phosphorylated on a tyrosine near the Stat COOH-terminus by a receptor-linked tyrosine protein kinase, usually Jak1-3, or Tyk2 [7], [9], [10]. After dissociation from the receptor docking site, Stats form dimers via reciprocal interactions of the Src homology 2 domain on one Stat molecule with the phosphorylated tyrosine on the other [9], and are translocated into the nucleus, where they bind as dimers to specific DNA sites in chromatin [7], [9]–[11]. Stats recognize the palindromic DNA sequence, 5′-TTCNxGAA-3′ (where N is any deoxynucleotide, and x = 2–4), but with distinct preferences depending on the individual Stat [9], [15].

Despite clear evidence that multiple signaling pathways act downstream of the GH receptor, recently identified inactivating molecular lesions in the STAT5B gene in humans with impaired growth [16], [17], targeted gene knockouts of the *GH receptor* [18], [19] and *Stat5b* in mice [20]–[22], and biochemical and molecular studies [23], have collectively implicated Stat5b as the essential signaling intermediate responsible for many of the critical biological actions of GH. For example, a key agent of GH-regulated somatic growth and tissue repair is insulin-like growth factor-I (IGF-I), a highly conserved 70-amino acid secreted protein [2], [24], whose gene transcription is rapidly and potently induced by GH via Stat5b [25], [26]. However, unlike most other genes whose transcription is acutely activated by GH through Stat5b, such as *Socs2, Cish,* and *Igfals* in rodents, in which functionally critical Stat5b binding sites are located within the proximal promoters, there are no Stat5b transcriptional response elements within either of the two promoters of the *Igf1* gene [27], [28]. Rather, several distinct GH-inducible Stat5b binding domains have been mapped to introns and to distal regions of human IGF-I and rat and mouse *Igf1* loci [29]–[34]. Although some of these elements appear to possess chromatin characteristics of transcriptional enhancers [34], their biochemical properties have not been elucidated to date.

Here we have evaluated the biochemical and functional characteristics of the multiple dispersed chromosomal Stat5b binding domains in the rat *Igf1* locus, as a means to understand how they contribute to control of IGF-I gene transcription by GH. We find that each Stat5b element has distinct transcriptional regulatory properties, and that individual sites within each element have unique binding profiles for Stat5b. Taken together, our data define a framework for discerning how Stat5b acts *in vivo* as the key mediator of GH-regulated IGF-I gene transcription.

**Table 1:** DNA Sequences of Oligonuceotide Primers for Cloning Stat5b Domains into *Igf1* Promoter 2 Reporter Plasmid.

Domain	Size (bp)	Location (bp from 5′ end of *Igf1*)^#^	Top Strand (5′ to 3′)*	Bottom Strand (5′ to 3′)*
R2–4	468	−86376	*BK*CCAAGACAATCCCCTGCATGCTAT	*BN*CCCTTTTGATTAATTGGGCTCAGG
R13	297	−63005	*XK*CTAAGATCCCCCTTGCTGATTTC	*XN*GGACGGAGTTCAGTTTTGACAC
R34–35	84	+3714	See Woelfle J, Chia DJ, Rotwein P (2003) J Biol Chem 278∶51261–51266	
R34–35	209	+3638	*XKL*ACCCTGTTGGTGACTCTTTCCA	*XN*AGCCAAATGACATCCCTGCCAA
R53–54	292	+26644	*BK*GGCACATGCCATTGACCAGATGATGTG	*BN*CTCTCTCCAAAAGAAATCTCCATTCACC
R57–59	208	+43721	*BLK*TATTCCTCCCAGCTGTGTGTCAC	*BN*TGGGACTTGGTCTGAGGCA
R60–61	241	+49028	*BK*AAGGGTTGCTGAGTGGTGGGGT	*BP*AGCTTGACCTTTGTCTTCTGAAAAAGTGC

#Refers to start of *Igf1* exon 1 on rat chromosome 7.

* *X* = GACTG (5′ overhang), *B* = GATC (5′ overhang), *H* = AAGCTT (HindIII site), *L* = GTCGAC (SalI site), S = GAGCTC (SacI site), *N* = GCTAGC (NheI site), *K* = GGTACC (KpnI site), *P* = CTGCAG (PstI site).

## Materials and Methods

### Materials

Fetal calf serum, Dulbecco's modified Eagle's medium, and phosphate-buffered saline were purchased from Mediatech-Cellgro (Herndon, VA). Transit-LT1 was from Mirus (Madison, WI), and the QuikChange site-directed mutagenesis kit from Stratagene (La Jolla, CA). Restriction enzymes, buffers, ligases, polymerases, and protease inhibitor tablets were from Roche Applied Sciences (Indianapolis, IN). Recombinant rat GH was purchased from the National Hormone and Pituitary Program, NIDDK, National Institutes of Health. Trypsin/EDTA solution was from Invitrogen (Camarillo, CA). The BCA and 660 nm protein assay kits were from Pierce Biotechnologies (Rockford, IL) and AquaBlock EIA/WIB solution from East Coast Biologicals (North Berwick, ME). QIA-Quick PCR purification kit was from Qiagen (Valencia, CA) and okadaic acid from Alexis Biochemicals (San Diego, CA). Primary antibodies were obtained from the following vendors: anti-phospho-Stat5 (clone 8-5-2) and anti-Creb, Millipore (Billerica, MA), anti-α−tubulin and anti-Flag (M2), Sigma-Aldrich (St. Louis, MO), anti-Stat5b, Invitrogen. Goat-anti-rabbit IgG-IR800 and goat anti-mouse IgG-IR680 were from Rockland Immunochemical (Gilbertsville, PA), and goat anti-mouse IgG1-Alexa 488 was from Invitrogen - Molecular Probes (Eugene, OR). Hoechst 33258 nuclear dye was from Polysciences (Warrington, PA). Oligonucleotides were synthesized at the OHSU DNA Services Core, at Oligos Etc (Wilsonville, OR), and at Eurofms MWG Operon (Huntsville, AL). All other chemicals were reagent grade and were purchased from commercial suppliers.

**Table 2:** DNA Sequences of Oligonucleotide Probes [core Stat5b binding site underlined].

Probe	Top Strand (5′ to 3′)	Labeled	Unlabeled
R2	CACCAATTCATGGAAATTAAAC		X
R3	AAAATATTTCCTGGAACTAAA		X
R4	CAAAGAATTTCTTCTTAGAATTTGTCAATTC		X
R13	CTTCCTTCCTTGAAACTG		X
R13.5	GAAACTGCCTTTTCCGTTGAATCTATCCTTCC		X
R34	GGGCCTTCCTGGAAGAAAG	X	X
R35	TCTGCTTCTTAGAATGAAG	X	X
R53	TCATCTTTCAGGGAAATCTAG		X
R54	GAATCCTTGTGTTTCTCTGAAATCCATAGCTAG		X
R57	AAGTTTTTCGAAGAATTGGAA		X
R58	TCCAGTTCTCAGAAAGGAA	X	X
R59	GGAAATTCGCAGAAGTGAG		X
R60	CCATGATTCCTAGAAAAGATGT		X
R61	CATAGTTCACAGAAAAGAGA		X

### Recombinant Plasmids

The following have been described previously: the expression plasmid in pcDNA3 for mouse GH receptor [23], [29], and reporter gene plasmids in pGL2 containing rat *Igf1* promoter 2 (*Igf1* P2-Luc and derivatives [34]). Flag-epitope tagged wild-type (WT), dominant negative (DN), and constitutively-active (CA) rat Stat5b also have been described [23], with point mutations being introduced to create DN Stat5b (Y699F) and CA Stat5b (N642H) using the Quik Change site-directed mutagenesis kit [23], [29]. The 84 base pair fragment containing R34–35 is described in Woelfle et al [29]. Additional reporter gene plasmids were generated by cloning 5′ to *Igf1* P2-Luc individual 208–468 base pair DNA segments amplified by PCR from rat genomic DNA that spanned Stat5b-binding domains from the *Igf1* locus. Point mutations were introduced into Stat5b sites using the QuikChange site-directed mutagenesis kit, as described [29]. All newly prepared DNA fragments in reporter plasmids and all engineered mutations were confirmed by DNA sequencing. Cloning primers are listed in Table 1.

**Figure 1:**
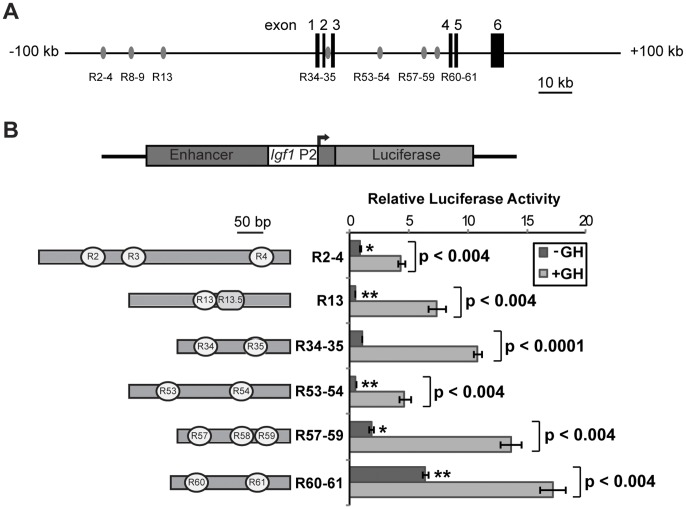
Stat5b binding elements in the rat *Igf1* locus confer GH-responsiveness to *Igf1* promoter 2 in promoter-reporter assays. A . Diagram of the rat *Igf1* locus showing seven conserved Stat5b binding elements, R2–4, R8–9, R13, R34–35, R53–54, R57–59, and R60–61. Each element is depicted as a gray oval and the six *Igf1* exons are shown as black boxes. **B**. Top - schematic of luciferase reporter plasmids containing rat *Igf1* promoter 2 (P2) and exon 2, and individual Stat5b binding elements (Enhancer). Bottom - Results of luciferase activity assays in Cos-7 cells transiently transfected with expression plasmids encoding the mouse GH receptor and rat Stat5b and reporter plasmids containing each putative enhancer region diagramed to the left (with each Stat5b site indicated as a white oval, and R13.5 as a gray curved shape), after incubation of cells with vehicle (dark bars) or rat GH [40 nM] (light bars) for 18 h. The graph presents results of 4 independent experiments for each promoter plasmid (mean ± S.E.; *, p<0.01; **, p<0.001 *vs.* R34–35 without GH [unpaired t-test]). Other p values are indicated, and compare ± GH treatment [paired t-test]. Luciferase counts for R34–35 without GH ranged from 3.5 to 5.5×10^3^ relative light units/sec.

### Cell Culture, Transient Transfections, and Reporter Gene Assays

Cos-7 cells (ATCC CRL-1651) were incubated in antibiotic-free Dulbecco's modified Eagle's medium with 10% fetal bovine serum at 37°C in humidified air with 5% CO_2_, and were transfected with expression plasmids for the mouse GH receptor and for wild-type Stat5b, Stat5b^CA^, or Stat5b^DN^ using Transit-LT1, as described [31]. The next day cells were incubated with rat GH [40 nM] or vehicle in serum-free medium and 1% bovine serum albumin, and nuclear and cytoplasmic protein extracts were prepared 1 or 2 h later [31]. Whole cell protein extracts were prepared after cells were incubated for 18 h in serum-free medium and 1% bovine serum albumin. For promoter-reporter assays, Cos-7 cells in 12-well dishes were co-transfected with mouse GH receptor (25 ng), Stat5b (100 ng), and individual promoter-reporter plasmids (187.5 ng), as described [34]. Twenty-four h later, cells were incubated for 16–18 h in serum-free medium and 1% bovine serum albumin ± rat GH [40 nM]. Cells were then harvested and lysates used for luciferase assays [34]. Results were normalized to total cellular protein concentrations.

**Figure 2:**
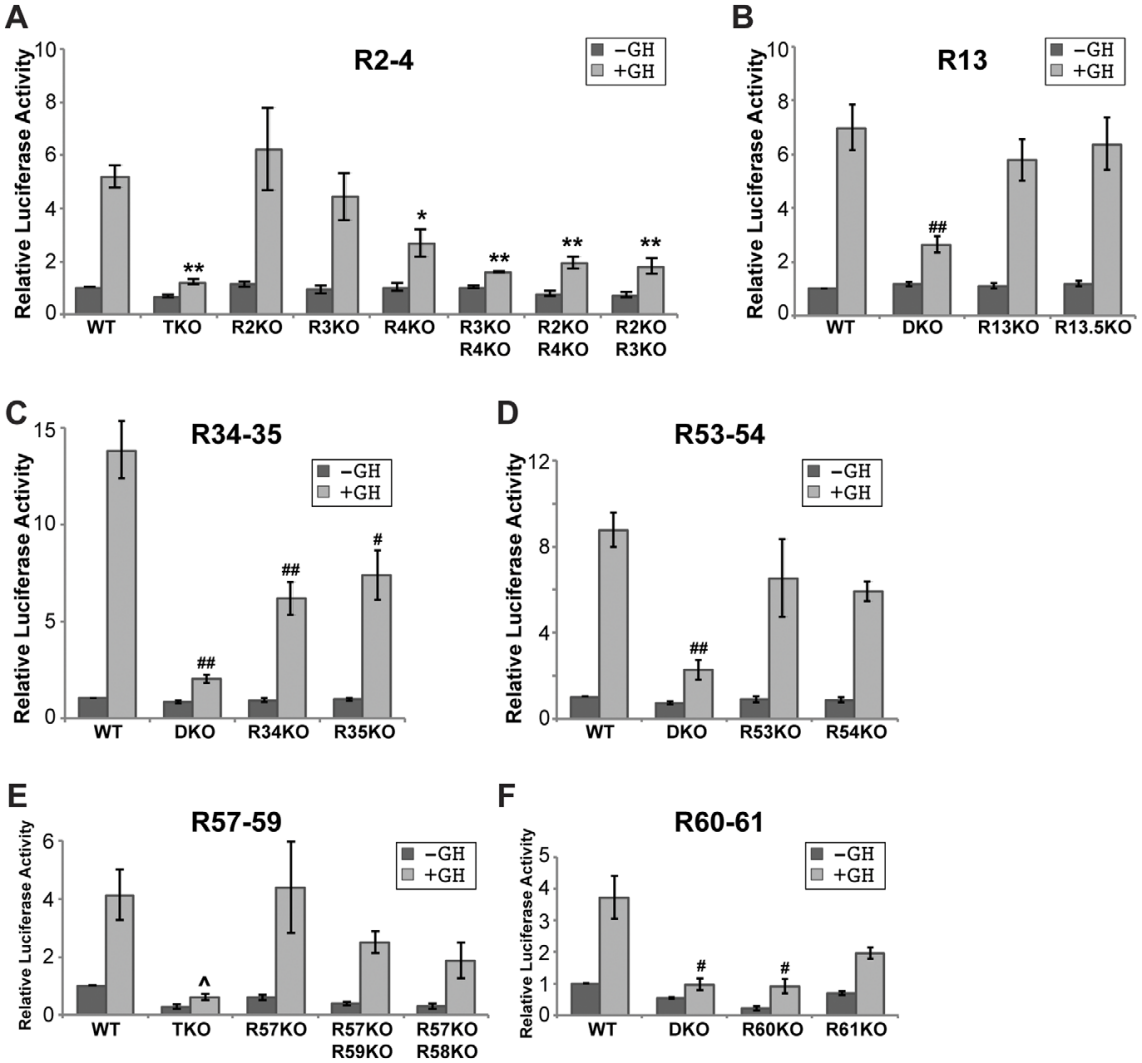
Stat5b binding sites are required to confer GH-responsiveness to *Igf1* promoter 2 in promoter-reporter assays. Results of luciferase assays in Cos-7 cells transiently transfected with reporter plasmids containing *Igf1* P2 and exon 2, plus wild type (WT) or mutated versions of individual Stat5b binding elements, and expression plasmids encoding the mouse GH receptor and rat Stat5b, and incubated with vehicle (dark bars) or rat GH [40 nM] (light bars) for 18 h. KO signifies knockout of a Stat5b binding site, with DKO representing double knockout and TKO, triple knockout. See ‘Materials and Methods’ for details. Bars represent the mean ± S.E. of 4–10 independent experiments (*, p<0.007; **, p<0.0007; #, p<0.017; ##, p<0.0017; ^∧^, p<0.013 *vs.* WT with GH [unpaired t-test]). In each graph, relative luciferase values obtained using the WT Stat5b element in the absence of GH were set to 1. **A**. R2–4. **B**. R13. **C**. R34–35. **D**. R53–54. **E**. R57–59. **F**. R60–61.

### Protein Immunoblotting

Isolation of nuclear and cytoplasmic proteins has been described previously [29], [31], [34]. Protein extracts were separated by SDS-polyacrylamide gel electrophoresis under denaturing and reducing conditions and transferred to 0.45 µM nitrocellulose membranes. Subsequent steps in immunoblotting were performed as described [31] with the following dilutions of primary antibodies: anti-Stat5b, 1∶5000, anti-phospho-Stat5, 1∶4000, anti-Flag, 1∶4000, anti-Creb, 1∶4000, anti-α-tubulin, 1∶10,000, and secondary antibodies at 1∶5000. Images were captured using the LiCoR Odyssey Infrared Imaging System (LiCoR, Lincoln, NE) and version 3.0 analysis software.

**Figure 3:**
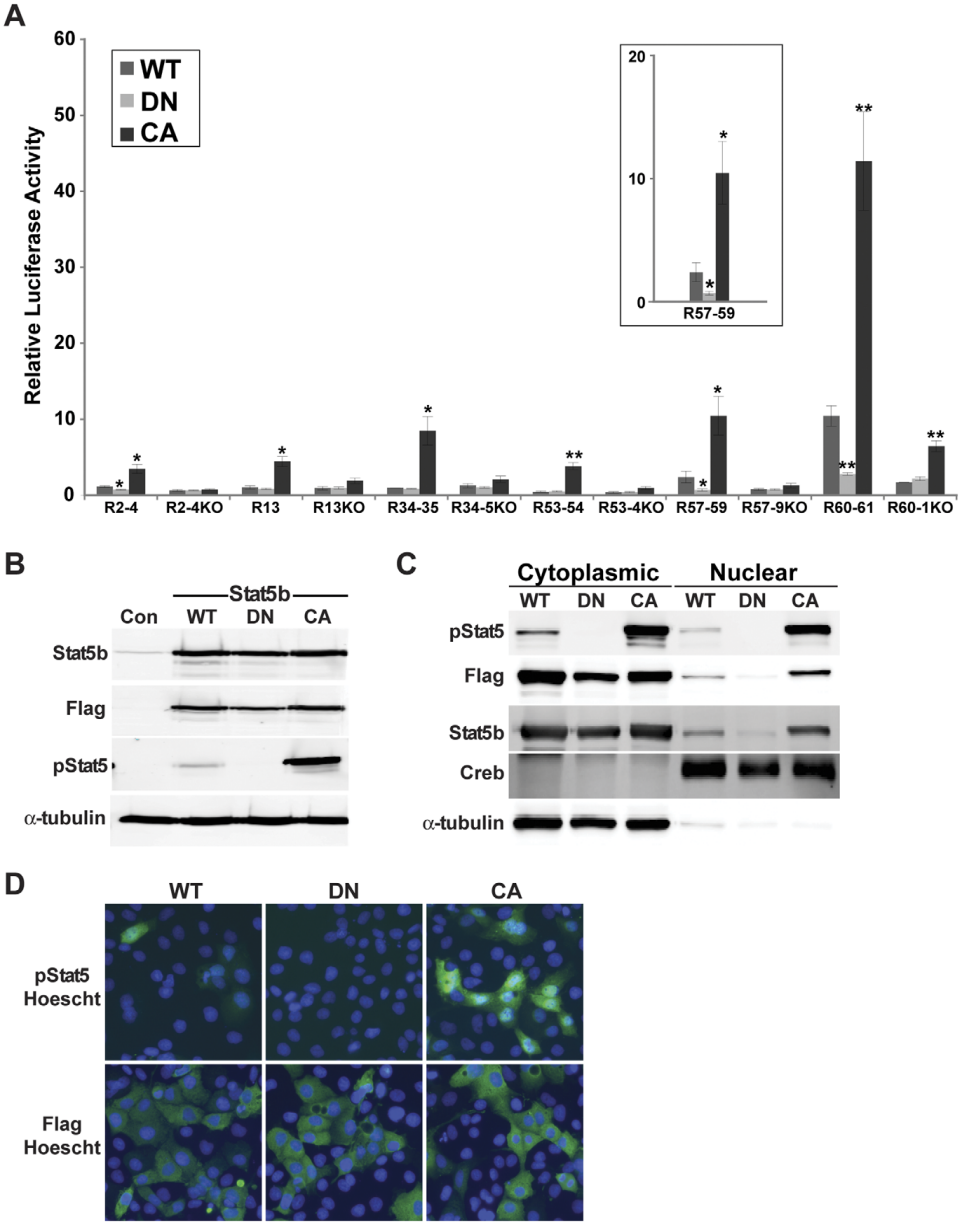
Stat5b differentially regulates the transcriptional activity of individual Stat5b elements in promoter-reporter assays. **A**. Results of luciferase activity assays in Cos-7 cells transiently transfected with expression plasmids encoding the mouse GH receptor and either wild type (WT), dominant negative (DN), or constitutively active (CA) rat Stat5b, and reporter plasmids containing *Igf1* P2 and exon 2, and each individual intact or mutated (KO) Stat5b binding element after incubation in serum free medium for 18 h. The graph depicts results of 4 independent experiments for each promoter plasmid comparing Stat5b^CA^ or Stat5b^DN^ with Stat5b^WT^ (mean ± S.E.; *, p<0.025; **, p<0.0025) [unpaired t-test]. The inset shows a higher power view of R57–59. **B**. Detection of Stat5b, the Flag epitope (for transfected WT, DN, and CA Stat5b), phospho (p) Stat5b, and α-tubulin by immunoblotting in whole cell protein extracts (Con = non-transfected control cells). **C**. Detection of Flag, pStat5b, Creb and α-tubulin by immunoblotting in nuclear and cytoplasmic protein extracts. **D**. Immunocytochemistry of Cos7 cells transfected with WT, DN, or CA Stat5b using antibodies to pStat5 (top) or Flag (bottom), after incubation in serum-free medium without GH for 18 h. Nuclei were stained with Hoescht dye (blue).

### Immunocytochemistry

Cos-7 cells in 6 well plates were transiently transfected with expression plasmids for wild type Stat5b, Stat5b^CA^, or Stat5b^DN^ (500 ng). Two days later, cells were fixed in 4% paraformaldehyde for 15 min at 20°C and permeabilized with a 50∶50 mixture of methanol and acetone for 2 min followed by blocking in 0.25% normal goat serum for 2 h at 20°C. After addition of Flag M2 monoclonal antibody (1∶2000 dilution) or pStat5 antibody (1∶2000 dilution) in blocking buffer overnight, followed by a washing step, and incubation in goat anti-mouse IgG_1_-Alexa 488 (1∶1000 dilution) in blocking buffer for 2 h, images were captured with a Nikon Eclipse E800 compound microscope with CCD camera, using Nikon analysis software. Nuclei were stained with Hoescht dye.

**Figure 4:**
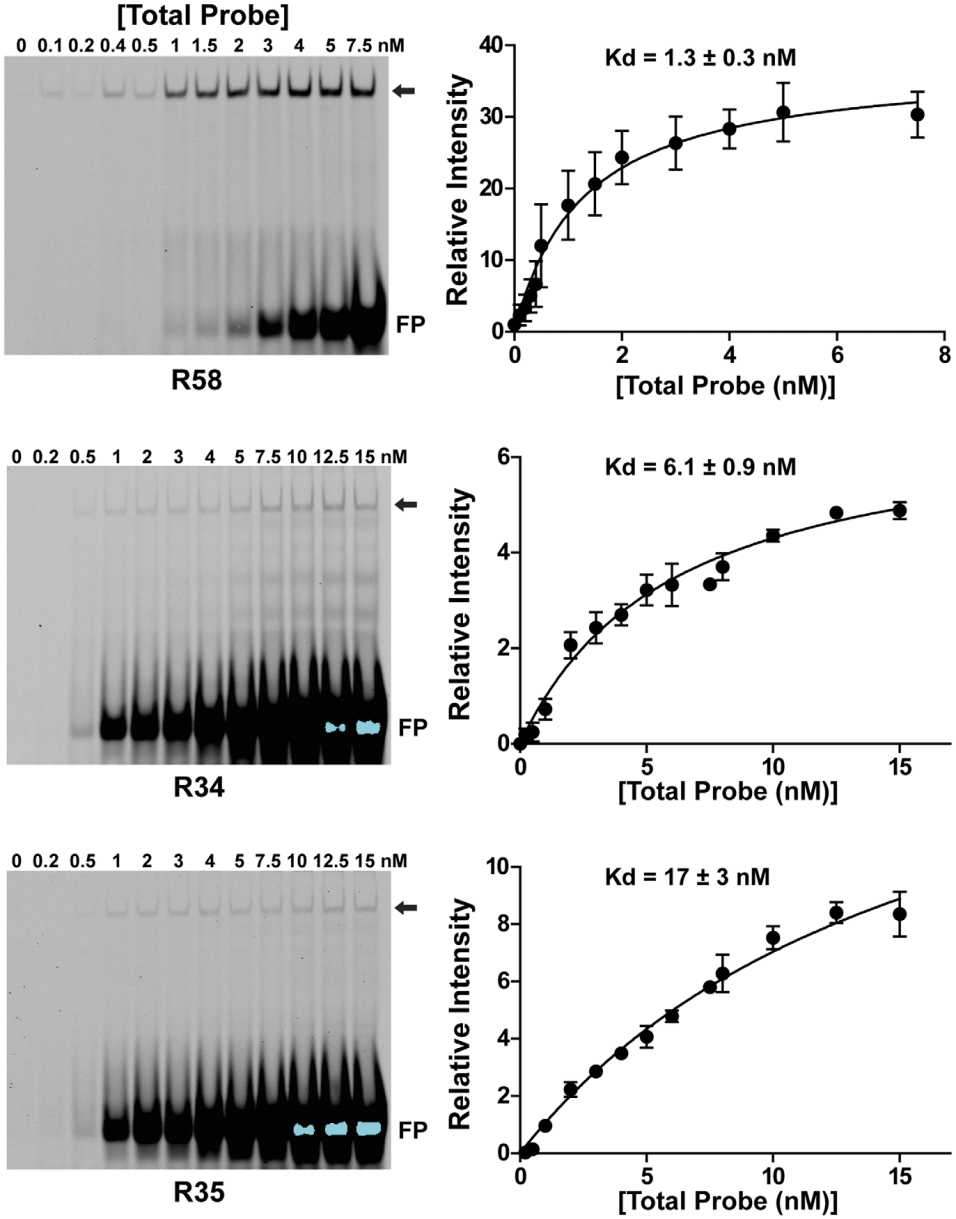
Assessing binding affinity of Stat 5b for individual DNA sites. Quantitative DNA-protein binding was assessed by gel-mobility shift experiments as described in ‘Materials and Methods’ using varying concentrations of Cy5.5-labeled double-stranded oligonucleotides for R58, R34, or R35, and 1 µg of nuclear protein from Cos-7 cells transfected with expression plasmids for the mouse GH receptor and wild-type rat Stat5b, and incubated with rat GH [40 nM] for 1 h. DNA binding was quantified with a LiCoR Odyssey infrared scanner and v3.0 analysis software, and results were plotted as shown. Left panels: representative results from individual experiments using nuclear proteins from cells expressing the mouse GH receptor and wild-type Stat5b after GH treatment. FP = unbound probe. The arrow indicates protein-DNA complexes. Right panels: binding curves with Kds listed (mean ± S.E., n = 3 experiments).

**Figure 5:**
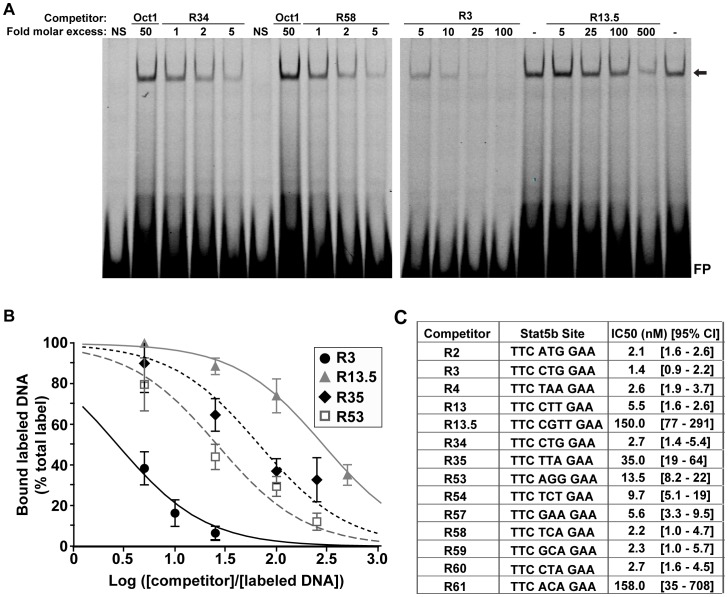
Defining a hierarchy of binding affinities of Stat5b for individual DNA sites within the rat *Igf1* locus. A . Gel-mobility shift experiments were performed with the Cy5.5-labeled double-stranded probe R34, 2 µg of nuclear protein from Cos-7 cells transfected with expression plasmids for the mouse GH receptor and rat Stat5b, and incubated with rat GH [40 nM] for 1 h, and various concentrations of competitor DNAs as indicated. Two representative individual competition experiments are shown. The arrow indicates the location of protein-DNA complexes (NS, no Stat5b in nuclear protein extract, FP = unbound probe). **B**. The graph illustrates results of competition experiments for 4 different unlabeled double-stranded competitor DNAs (mean ± S.E., n = 3 independent experiments, with 4 data points/experiment). **C**. Results for all probes have been tabulated (n = 3 independent experiments, with 4 data points/experiment) and are presented as IC50 values (DNA concentration at which binding of labeled probe is reduced to 50% of starting value). The 95% confidence interval (CI) also is indicated and each Stat5b core DNA binding sequence is listed.

### DNA-protein Binding Studies

Electrophoretic gel-mobility shift assays and DNA competition experiments were performed as described [31] with Cos-7 nuclear protein extracts and 5′-Cy5.5-labeled double-stranded oligonucleotides. Sequences for the top strand for all double-stranded DNA probes tested for binding by Stat5b are listed in Table 2. The top strand of the Oct-1 probe is as follows (core binding site is underlined): 5′-TTTTAGAGGATCCATGCAAATGGACGT ACGT-3′. After incubation of proteins and DNA for 60 min at 4°C, products were separated by electrophoresis through non-denaturing 5% polyacrylamide gels in 1× Tris borate/EDTA (90 mM Tris, 90 mM boric acid, 2 mM EDTA, pH 8.3) at 200V for 30 min at 20°C. After electrophoresis, gels were dried and the bands representing protein-bound DNA and free probe measured using the LiCoR Odyssey and version 3.0 analysis software. Quantitative DNA-protein binding studies were performed as described [31] with a constant amount of protein (1 µg of Cos-7 cell nuclear extract), and thus a constant quantity of transcription factor, and varying concentrations of 5′-Cy5.5-labeled probes [0.1 to 15 nM]. Dissociation constants (Kd) were calculated assuming a model of single-site specific binding, using Prism 5.01 for Windows (GraphPad Software, La Jolla, CA) to perform a least-squares non-linear fit of the saturation binding curves. Competition experiments employed nuclear protein extracts (2 µg) and varying amounts of unlabeled homologous and heterologous competitor DNAs. Calculations of dissociation constants for competitors (IC50) were performed with Prism 5.01 for Windows, using a single-site model that constrained the maximal and minimal “bound” values.

**Figure 6:**
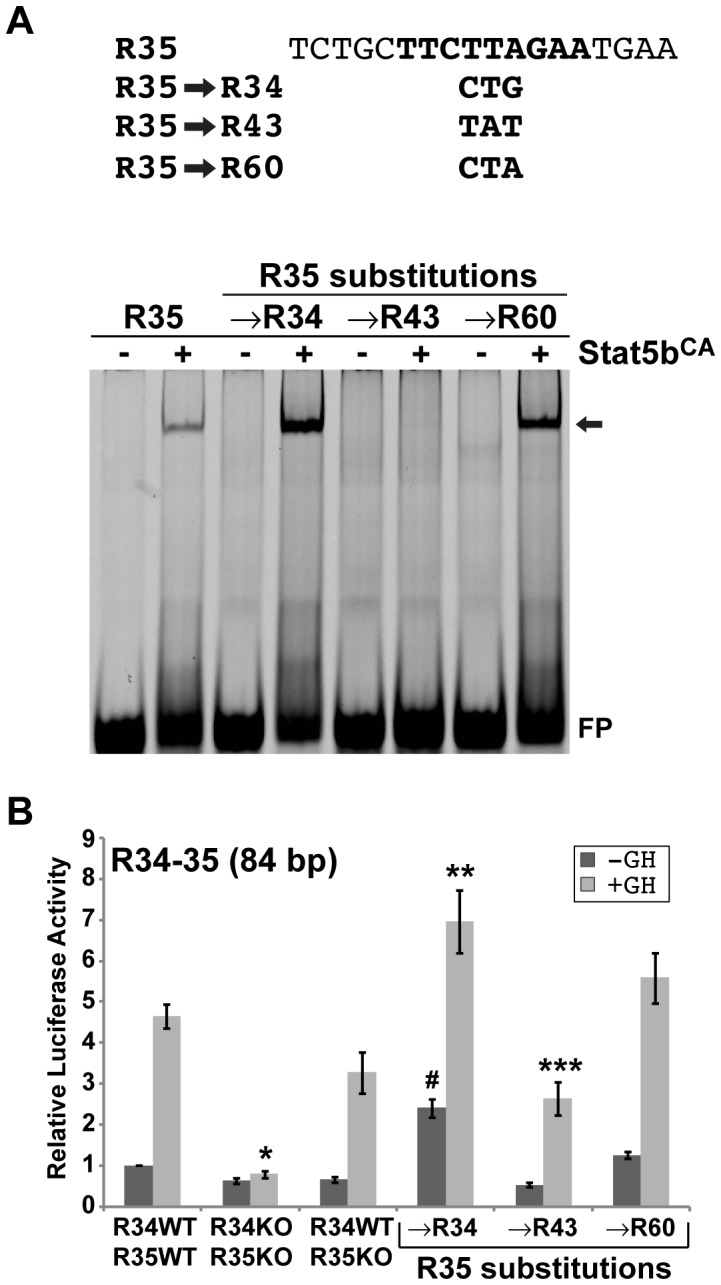
DNA modifications in the core Stat5b recognition sequence alter binding and Stat5b-dependent transcriptional activity. A . Top: Nucleotide sequence of top strand of DNA probe of R35 used in gel-mobility shift experiments. The 9-base pair central Stat5b recognition site is in bold script. Nucleotide substitutions to create modified DNA probes are listed below. Bottom: Results of gel-mobility shift assays with IR-labeled double-stranded oligonucleotide probes and 2 µg of nuclear protein from Cos-7 cells transfected with empty vector (−) or with expression plasmid for rat Stat5b^CA^ (+). The probes are labeled above each pair of lanes. The arrow indicates protein-DNA complexes. FP = unbound probe. **B**. Results of luciferase assays in Cos-7 cells transiently transfected with reporter plasmids containing *Igf1* P2 and exon 2, plus 84 base pair wild type (WT) R34–35, or with modified Stat5b sites as indicated, and expression plasmids encoding the mouse GH receptor and rat Stat5b, and incubated with vehicle (dark bars) or rat GH [40 nM] (light bars) for 18 h. KO – knockout of binding site. Bars represent the mean ± S.E. of 6 independent experiments (*, p<0.000001; **, p<0.009; ***, p<0.0004 *vs.* WT with GH; #, p<0.00001 *vs.* WT without GH [unpaired t-test]).

### Statistical Analysis

Data are presented as mean±S.E. Statistical significance was determined using either paired or unpaired Student’s *t* test, with the Bonferroni correction for multiple comparisons in the latter, and is indicated in each figure legend.

## Results

### Stat5b Binding Elements Confer GH Responsiveness to *Igf1* Promoter 2

In previous studies we used a combination of bioinformatics and chromatin immuno-precipitation experiments to identify 7 distinct regions in the rat *Igf1* locus that exhibited acute GH-stimulated binding of Stat5b in hepatic chromatin [34], and found in preliminary studies that all of these domains except for R8-9 could enhance the activity of *Igf1* promoters in a GH- and Stat5b-dependent way [34]. We now have investigated the functional and biochemical properties of these elements in detail as a means of elucidating the mechanisms by which they contribute to regulation of IGF-I gene transcription. The overall anatomy of the rat *Igf1* locus is depicted in Fig. 1A, with the location of each of the putative enhancer domains indicated; a higher power view of their individual organization is illustrated in Fig. 1B. With the exception of R13, each of the 6 elements tested encodes two or three *bona fide* Stat5 binding sites, each consisting of the DNA sequence, 5′-TTC NNN GAA-3′ (top strand, where N = G, A, T, or C), with individual sites being separated by 6–251 base pairs of genomic DNA (Fig. 1B). Immediately adjacent to R13 is the DNA sequence, 5′-TTC CGTT GAA-3′ (top strand, and labeled R13.5 in Fig. 1B), which conforms to an optimal binding site for Stat6 [15].

Functional studies were performed by reconstituting GH-activated signaling in cultured cells by expressing the mouse GH receptor and rat Stat5b, and using as transcriptional reporter genes recombinant plasmids containing a minimal 80 base pair fragment of proximal rat *Igf1* promoter 2 plus 44 base pairs of adjacent exon 2 fused to luciferase, with each Stat5b element being cloned 5′ to the promoter (Fig. 1B). As seen in Fig. 1B, each of the 6 Stat5b domains tested significantly boosted *Igf1* promoter function in Cos-7 cells after GH treatment (from 2.8 to 12-fold). Previous studies had found that no response to GH was seen in the absence of added Stat5b expression plasmid [29].

To test the hypothesis that binding of Stat5b was necessary for GH-activated transcription mediated by these genomic DNA segments, point mutations were engineered into individual Stat5b sites in each of the six elements by changing 5′-TTC N_3–4_ GAA-3′ to 5′-**G**TC N_3–4_ G**T**A-3′ (modifications are in **bold** script). Results from promoter-reporter studies demonstrated that in all cases elimination of every Stat5b site in a genomic fragment markedly attenuated GH- and Stat5b-activated transcription (Fig. 2A–F, double (D) knockout (KO), triple (T) KO). However, the impact of loss of individual Stat5b sites varied within each element. For example, removal of either R34 or R35 reduced transcriptional activity by ∼50% (Fig. 2C), while loss of R53 or R54 caused only a ∼25% decline, and elimination of R13 or R13.5, only ∼15% (Fig. 2D and 2B, both statistically not significant). In contrast, loss of R60 alone was as effective as double elimination of R60 and R61 (Fig. 2F).

Analysis of the two transcriptional elements with 3 Stat5b binding sites gave a more complicated picture. For R2–4, individual loss of R4 but not R2 or R3 led to a significant decline in responsiveness of *Igf1* promoter 2 to GH, although each double elimination reduced transcriptional activity nearly as effectively as the triple knockout (Fig. 2A). For R57–59, removal of R57 had no effect, and only the triple deficiency among the combinations tested caused a statistically meaningful reduction in reporter gene function (Fig. 2E).

### Selective Activity of Stat5b on Individual *Igf1* Locus Enhancer Elements

Inspection of the data in Fig. 1B showed that there were substantial differences in transcriptional activity in the absence of GH depending on which Stat5b element was fused to *Igf1* promoter 2. For example, the basal values of a luciferase reporter plasmid with R60–61 were 8-times higher than one containing R53–54 or R13–13.5 (Fig. 1B). To test the hypothesis that ‘inactive’ Stat5b could either differentially activate or inhibit target gene transcription via individual Stat5b responsive elements, studies were performed in the absence of GH, using expression plasmids encoding either previously-validated wild type (WT), dominant-negative (DN), or constitutively-active (CA) Stat5b [31], and *Igf1* promoter 2 - reporter genes containing individual intact enhancers or enhancers in which all Stat5b binding sites were disrupted by point mutations. For 4 of the native enhancer - promoter - reporter plasmids tested, ‘inactive’ Stat5b^WT^ and Stat5b^DN^ had little differential effect on gene transcription, although in all cases Stat5b^CA^ was stimulatory by 3-8-fold (Fig. 3A, R2–4, R13, R34–35, R53–54). The exceptions were R57–59 and R60–61, in which ‘inactive’ Stat5b^WT^ was able to drive promoter function to 3–4-fold higher levels than Stat5b^DN^, although only to ∼25% of the values obtained with Stat5b^CA^ (Fig. 3A). This apparent stimulatory effect of wild type Stat5b was lost in cells expressing *Igf1* promoter 2 - reporter genes in which the Stat5b binding sites were destroyed (KO, Fig. 3A). Similarly, the transcriptional response to Stat5b^CA^ was attenuated in all reporter genes with mutated enhancer elements (Fig. 3A). Levels of expression of transfected Stat5b^WT^, Stat5b^DN^, and Stat5b^CA^ were nearly identical (Fig. 3B), but examination of their sub-cellular location in the absence of GH treatment showed that Stat5b^CA^ was found in the cytoplasm and nucleus and was tyrosine phosphorylated, that Stat5b^DN^ was in the cytoplasm, and that a small amount of Stat5b^WT^ was nuclear and tyrosine phosphorylated (Fig. 3C and D). Taken together, these results demonstrate a selective transcriptional stimulatory effect of Stat5b on 2 of 6 Stat5b-responsive enhancers in the absence of GH-induced activation, implying that individual *Igf1* locus Stat5b-regulated responsive elements have different functional properties.

### DNA Binding Strength and Transcriptional Function

Quantitative *in vitro* DNA-protein binding experiments [31] revealed a ∼15-fold difference in affinities of GH-activated wild-type Stat5b for the 3 different Stat5 sites studied with this method: R58, R34, and R35 (Fig. 4, Kd values from 1.3 to 17 nM). Similar results were observed with cells expressing Stat5b^CA^, although the range of affinities was narrower (Kds from 3.2 to 8.5 nM, data not shown). Analyses using semi-quantitative dose-response competition studies against the labeled R34 probe allowed us to calculate relative affinities of GH-activated wild-type Stat5b for each of the 14 Stat5 sites in the 6 elements. Results show a wide range of binding strengths, as illustrated by measured IC50’s of 1.4 to 158 nM (Fig. 5). Of note, R13.5, which resembled an optimal Stat6 site [15], competed very poorly for binding of Stat5b to the labeled R34 probe (Fig. 5A, C), thus indicating that despite its clear contribution to Stat5b-mediated transcription (Fig. 2), at best it could bind Stat5b very weakly. Also of note are close correlations between binding affinities determined directly with wild-type Stat5 and those measured by competition studies (R58, Kd of 1.3 nM, IC50 of 2.2 nM; R34, Kd of 6.1 nM, IC50 of 2.7 nM; R35, Kd of 17 nM, IC50 of 35 nM).

We next examined the direct impact of substituting one Stat5 site for another on both DNA binding affinity and transcriptional activity. Changing low affinity R35 to either higher affinity R34 or R60 within the 18 base pair R35 oligonucleotide probe caused a substantial increase in binding strength of Stat5b^CA^ as revealed by gel-mobility shift experiments (Fig. 6A), along with a commensurate rise in GH-activated and Stat5b mediated transcription of *Igf1* promoter 2 fused to modified R34–35 (Fig. 6B, although this did not quite reach statistical significance for R60). Of note, promoter activity also was increased by nearly 4-fold in the absence of GH with the R35 to R34 substituted DNA (Fig. 6B). In contrast, substitution with R43, which does not bind Stat5b *in vitro* or *in vivo* [34], eliminated binding by qualitative EMSA (Fig. 6A) and reduced GH-activated transcriptional activity, with the latter being equivalent to knockout of the R35 site (Fig. 6B). Taken together, the data in Fig. 6 show that 1 or 2 nucleotide modifications within a single Stat5b site that result in a change in binding affinity can alter the functional properties of the entire enhancer element.

## Discussion

Gene targeting experiments in mice and the identification and analysis of several natural mutations in humans have established unequivocally that the transcription factor Stat5b is a critical mediator of somatic growth via the GH - IGF-I axis [16], [17], [20], [21]. Molecular and biochemical studies have determined that the hormone-stimulated GH receptor activates Stat5b through the receptor-associated tyrosine kinase, Jak2 [1], [8], leading to rapid induction of IGF-I gene transcription [23], [29]. Unlike other GH- and Stat5b-regulated genes, IGF-I lacks Stat5b response elements in its proximal promoters [34], and it has been postulated based on studies of rat and mouse *Igf1* that GH- and Stat5b-stimulated IGF-I gene transcription occurs through the recruitment of multiple distinct GH-regulated Stat5b binding elements in chromatin with potential characteristics of long-range transcriptional enhancers [34]. Here we have dissected the biochemical properties of six of these Stat5b-responsive chromosomal domains. Our results show that these elements contain a number of remarkable shared and individually distinctive properties.

Using a series of promoter-reporter gene studies, in which individual Stat5b-binding domains were fused adjacent to *Igf1* promoter 2 in a reconstituted GH- and Stat5b-dependent cell system, we first established that all six elements analyzed encode at least 2 functionally important Stat binding sites. With the exception of the domain R60–61, where loss of R60 inhibited GH-stimulated transcription as effectively as the double elimination of R60 and R61, all individual sites appear to be required for full hormonal responsiveness (Fig. 2). In a recent publication, where we first identified multiple Stat5b binding elements within the rat *Igf1* locus [34], we suggested that R13 was an outlier, as it contained only a single Stat5b site. We now find that the DNA sequence 5′-TTC CGTT GAA-3′, a canonical site for Stat6 [15], located 8 base pairs 3′ to R13, is essential for full transcriptional activity of the enhancer, even though this DNA sequence does not compete effectively for binding of Stat5b in gel-mobility shift experiments, and at best binds Stat5b directly with low affinity. Our observations are consistent with results of two whole-genome screening studies for interactions of Stat5 with chromatin, which have found that the majority of transcriptional regulatory elements contain paired Stat5 binding sites [35], [36], although single sites and non-canonical sequences were identified in a more recent publication [37].

Our second major observation relates to the potential differential responsiveness of the R57–59 and R60–61 elements to Stat5b^WT^ compared with the other 4 Stat5b binding domains. We found surprisingly that in the absence of GH-stimulated activation Stat5b^WT^ boosted the transcription of *Igf1* promoter 2 when linked to R57–59 or R60–61 but had no effect on the other Stat5b-regulated enhancers (Fig. 3). Since the ability of Stat5b^WT^ to selectively induce *Igf1* promoter activity under the experimental conditions of no GH treatment was curtailed when the Stat5b binding sites in the R57–59 and R60–61 were eliminated, our results indicate that individual Stat5b-binding transcriptional enhancers in the *Igf1* locus have distinct physiological properties, with R57–59 and R60–61 being more sensitive than the other enhancers tested. These functional differences between R57–59 and R60–61 and the other 4 elements did not correlate fully with *in vitro* binding affinities for Stat5b, since, for example, R2–4, which was not activated by Stat5b^WT^ in the absence of GH, contains higher affinity sites than R57–59. Despite the fact that our results show that R57–59 and R60–61 have different functional properties than the other GH-regulated Stat5b domains in the *Igf1* locus, they do not reveal how these Stat5b binding enhancers either individually or collectively control *Igf1* gene transcription in chromatin.

Although it was thought previously that latent Stats reside in the cytoplasm [9], several Stats, including Stat5a, have been shown to undergo continuous shuttling between cytoplasmic and nuclear compartments in the absence of activation by cytokines [38], [39], and it has been found that nuclear retention is enhanced by acquisition of DNA binding capability, which follows cytokine-mediated tyrosine phosphorylation [38]. Even though it has not been established definitively that Stat5b undergoes continuous cytoplasmic - nuclear shuttling, it is 96% identical to Stat5a, including 98–99% identity in the ∼192 amino acid coiled-coil domain [40], which is the region of Stat5a critical for constitutive nuclear import [39]. Of note, our detection of wild type Stat5b in nuclear protein extracts of transiently transfected Cos-7 cells in the absence of its GH-mediated activation (Fig. 3C and D) supports the idea that Stat5b can undergo shuttling.

Our third observation also relates to the apparently complex relationship between binding of Stat5b to individual DNA sites and the transcriptional competence of domains encoding two or three sites. Results from quantitative and semi-quantitative gel-mobility shift experiments revealed a wide range of binding affinities (spanning 2 orders of magnitude, Figs. 4 and 5), and there appeared to be no more than a rough correlation between affinities of Stat5b for individual sites within a domain and overall transcriptional activity of that DNA segment or its responsiveness to GH-activated Stat5b. For example, the transcriptional impact of GH on an *Igf1* promoter fused to R13–13.5, which contains two weak Stat5b binding sites, was twice that of R53–54, which encodes two higher affinity sites, and nearly twice that of R2–4, which contains three high affinity sites, but was half of R60–61, which has one high and one very low affinity site (compare Fig. 5 and Fig. 1). Taken together, these data suggest that other features of individual GH- and Stat5b-responsive elements in addition to the Stat5b binding sites control their potency as GH-regulated transcriptional enhancers, and indicates that additional studies will be needed to dissect these enhancers fully. However, in the individual R34–35 domain, when a higher affinity site (R34, R60) replaced lower affinity R35, not only did *in vitro* binding of Stat5b increase, but so did the GH-mediated transcriptional response of the entire R34–35 element (although this did not quite reach statistical significance for R60). Similarly, when R35 was replaced by R43, which does not bind Stat5b [34], DNA binding was abrogated and transcriptional activity was impaired. As each of these changes involved only one or two nucleotides within an 18 base pair probe or within an 84 base pair enhancer element (Fig. 6), the results demonstrate dramatic specificity and sensitivity in the ability of Stat5b to read DNA binding activity and transform it into transcriptional function.

GH orchestrates rapid and dramatic alterations in gene expression to yield potent biological effects on growth, metabolism, and tissue repair [1], [2], [26], as well as exerting longer-term actions with potential pathogenic impacts on aging and on carcinogenesis [3]–[6]. The key role of Stat5b in mediating changes in gene expression in response to GH is now clearly established, yet our understanding of how this potent transcription factor powerfully regulates critical GH-target genes such as IGF-I will require a more comprehensive elucidation of its biochemical and molecular mechanisms of action. Studies in relevant experimental models are needed to determine if interplay in chromatin among multiple enhancers with the two IGF-I promoters collectively regulates IGF-I gene activity under different physiological situations.
